# An Interesting and Rare Case

**Published:** 1890-09

**Authors:** L. P. Blair


### AN INTERESTING AND RARE CASE.

BY L. P. BLAIR, D.D.S.

Miss T., a young lady, twenty-six years old, came to me with a very peculiar condition of face. The portion which extends from median line of lips to angle of the mouth and from infraorbital canal to mylo-hyoid ridge, was entirely gone. She desired to know if I could do anything for her.

The history of the case is this. Up to the age of six years she was perfectly healthy; at that time she was taken with typhus-pneumonia. The physician in attendance gave calomel and quinine, forty-six hours after which a blue spot was noticed on the inside of the cheek; the next morning it was visible on the outside of the face, and before night the portion of her face extending from the eye to condyle of jaw along the mylo-hyoid ridge and median line of face developed a line of sloughing, and in a few hours entirely dropped out, exposing the bones. The temporary teeth dropped out and the motion of jaw ceased and became ankylosed.

The young lady had had two attempts made with plastic operations, both of which were only partially successful. When she applied to me the lateral, canine, and two bicuspid teeth, in the left superior maxillary, had been removed in order to allow her to eat, drink, and articulate. For twenty years she had lived eating through that small aperture.

She wanted to know if something could not be done to hide that ugly gap. I told her I thought it possible. First I took a plaster cast of the face and moulded paraffine and wax upon it until I had the face as near perfect as I could get it. I then took a cast of the face and obtained my die; I next placed sheet-wax upon this and forced it into position, thus getting a skeleton form; then trying it upon the face, I found it to be correct. I subsequently made two crowns to fit the lower bicuspids and placed them in position; then two bands to fit upon the crowns in the form of spring clasps, leaving an arm from each extending through the wax form. These arms I securely fastened and removed the form with clasps in position. Having now the model I desired, I invested in plaster and proceeded as in plate-work. I rough-finished and sent to artist to be painted. The artificial portion of the face was made of pink and brown rubber painted. I simply filed off the outside, polishing thoroughly the inside.

The result is, the young lady has a false part that so nearly resembles the other side of the face that none but the very observing would notice any difference. This fixture obstructed the aperture for feeding; to obviate this difficulty I removed the right centrals, laterals, and canines in upper and lower jaw, thus allowing her to eat, drink, and articulate without removing the appliance. She has now been wearing it five months with perfect satisfaction. The illustrations show the face before and after the operation.

